# Graphene and Polymer Composites for Supercapacitor Applications: a Review

**DOI:** 10.1186/s11671-017-2150-5

**Published:** 2017-06-02

**Authors:** Yang Gao

**Affiliations:** Department of Electrical and Computer Engineering, University of California, Davis, CA 95616-5294 USA

**Keywords:** Graphene, Polymers, Composites, Supercapacitors, Flexible

## Abstract

Supercapacitors, as one of the energy storage devices, exhibit ultrahigh capacitance, high power density, and long cycle. High specific surface area, mechanical and chemical stability, and low cost are often required for supercapacitor materials. Graphene, as a new emerging carbon material, has attracted a lot of attention in energy storage field due to its intrinsic properties. Polymers are often incorporated into graphene for a number of enhanced or new properties as supercapacitors. In this paper, different polymers which are used to form composite materials for supercapacitor applications are reviewed. The functions, strategies, and the enhanced properties of graphene and polymer composites are discussed. Finally, the recent development of graphene and polymers for flexible supercapacitors are also discussed.

## Introduction

Since its discovery in 2004, graphene, one atom thick single carbon layer, is one of the most studied materials today. The fundamental properties of graphene made it very promising in a number of applications such as electronics, mechanics, optics, etc. In particular, graphene sheet has a theoretical specific surface area of 2630 m^2^/g [1], which has attracted great interest in energy storage applications including supercapacitors and batteries. The specific surface area of graphene is much larger compared to black carbon (typically <900 m^2^/g) and carbon nanotubes (from 100 to 1000 m^2^/g), but similar to activated carbon [2]. Today, supercapacitor manufacturers mostly use activated carbon made from coconut shell as the active material in their supercapacitor electrodes, because of its high specific surface area, cheap price as well as the capability of mass production. Activated carbon used in supercapacitor applications is a premium grade carbon which is purified to reduce ash, halogen, iron, and other impurities to less than 1% to enable extended cycling of devices. Over time, the cost of activated carbon has dropped from $150–200/kg to $15/kg, and this low price is a formidable barrier for other carbon materials to enter the market [3]. Graphene sheet which is grown via chemical vapor deposition (CVD) directly on copper or nickel foil [4] has the highest quality and the least defects. However, it would not be a good candidate to compete against the activated carbon for supercapacitor applications, since it is too expensive to manufacture and it is hardly scalable. Today, most studies on graphene-based supercapacitors focus on graphene nanoplatelets, graphene nanopowders and other graphene derivatives such as graphene oxide, reduced graphene oxide, chemical modified graphene, etc. These forms of graphene normally consist of double or multiple stacks of graphene nanosheets. Unlike graphene from CVD process, these forms of graphene are made by relatively cheap chemical, mechanical, or thermal exfoliation process of graphite. There are more surface defects in these graphene derivatives compared to their CVD graphene counterpart, and this may prevent them to be used as high-end electronic, photonic/optoelectronic devices such as transistors, photodetectors, large-scale transparent conducting electrodes, etc. However, the increased density of surface defects is more favorable for supercapacitor applications and often lead to an increase in electrochemical capacitance capability [5]. In addition, the cost is one of the most important factors to consider for any practical supercapacitor device. These graphene nanoplatelets have high surface area and are often used as the active materials of the supercapacitor electrode.

## Review

### Graphene and Polymer Binders

In order to bind graphene nanosheets onto the current collector, polymer binders are often needed. Among them, fluoropolymers such as polyvinylidene fluoride (PVDF) and polytetrafluoroethylene (PTFE) are mostly used.

#### Graphene and PVDF Supercapacitors

PVDF is a highly nonreactive thermoplastic fluoropolymer that exhibits high mechanical strength, good chemical resistance, thermal stability, and excellent aging resistance [6]. It has a variety of applications such as chemical, semiconductor, medical materials, and lithium ion batteries [6, 7]. For graphene-based supercapacitors, PVDF is mainly used as a binder material to bind graphene nanoplatelets or nanopowders onto the current collector as well as maintaining the electrode feature and providing mechanical strength. To form a graphene-based supercapacitor electrode, graphene nanoplatelets and 10–20 wt% of PVDF are mixed first. To achieve a good mixing result, it usually needs to knead PVDF and graphene nanoplatelets into a paste-like composite, and then adjust the viscosity by adding organic solvents such as N-Methyl-2-pyrrolidone (NMP) or dimethylformamide (DMF) to form a slurry. The composite slurry is coated on the current collector and then dried and compressed to form the supercapacitor electrode. There are several coating techniques such as doctor blade coating, bar coating, drop cast, etc.

PVDF content has to be precisely controlled since insufficient PVDF content does not provide enough binding strength between the active graphene and the current collector and can lead to an adhesion issue. On the other hand, too much PVDF content reduces the conductivity of the supercapacitor electrode and thus reduces the energy density and power density of a supercapacitor. To minimize the adverse conductivity effect of PVDF, a certain percentage of conductive carbon such as carbon black (CB), acetylene black, etc. can be introduced to the mixture. It is reported that conductive carbon nanoparticles can (i) act as a filler material to suppress the aggregation of graphene nanosheets, (ii) improve rate capacity and cycle stability of the supercapacitor electrode, and (iii) maintain the high electrical conductivity of the electrode [8]. These conductive carbon materials are normally 20–50 nm in size [9], and are dispersed evenly onto graphene material. In [9], the active materials were made of 90% of reduced graphene and 10% of CB, and the active materials were mixed with PVDF with a ratio of 95:5 and then coated onto nickel foam electrode. The addition of CB does not change the overall morphology of the reduced graphene/PVDF electrode. A specific capacitance of 175 Fg^−1^ and only 9.1% capacitance decrease over 6000 cycles of the reduced graphene/PVDF/CB supercapacitor electrode were obtained in [9].

**Fig. 1:**
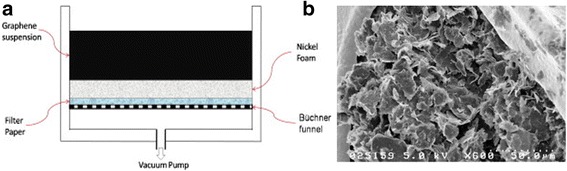
**a** Schematics of vacuum filtration method to build graphene-containing supercapacitor electrode. **b** SEM image of graphene/PVDF on the nickel foam formed by vacuum filtration. Reprinted with permission from [10]. Copyright 2012 Elsevier

In addition to mixing and coating method, vacuum filtration method can also be employed to form the graphene/polymer supercapacitor electrode onto a porous current collector such as nickel foam. The schematics of the vacuum filtration are shown in Fig. 1a [10]. The amount and the distribution of the graphene can be adjusted by vacuum pressure and process duration. In [10], graphene nanoplatelets were used as the active material, 25 wt% of PVDF as the binder material and a 95% porosity nickel foam as the electrode. The SEM image of the as-deposited graphene-containing electrode formed by vacuum filtration is shown in Fig. 1b. A specific capacitance of 152 Fg^−1^ and 95% capacitance retention over 2000 cycles were reported using this method.

#### Graphene and PTFE Supercapacitors

PTFE is also a commonly used supercapacitor polymer binder, and it is another type of fluoropolymer similar to PVDF. Unlike PVDF, it has more fluorine atoms on its backbone. Another difference lies in the solubility in water. PVDF is usually in white powder form and needs organic solvent such as NMP to mix with the active materials (e.g., activated carbon, graphene, and carbon nanotubes). However, PTFE can be dispersed in water (e.g., PTFE 60 wt% dispersion in H_2_O) [11–13], isopropanol [14, 15], and ethanol [16–18]. Activated carbon supercapacitor industry is inclined to using PTFE due to this feature as it is restively cost efficient to handle water solvent not to mention the safety-related issues of using an organic solvent.

**Fig. 2:**
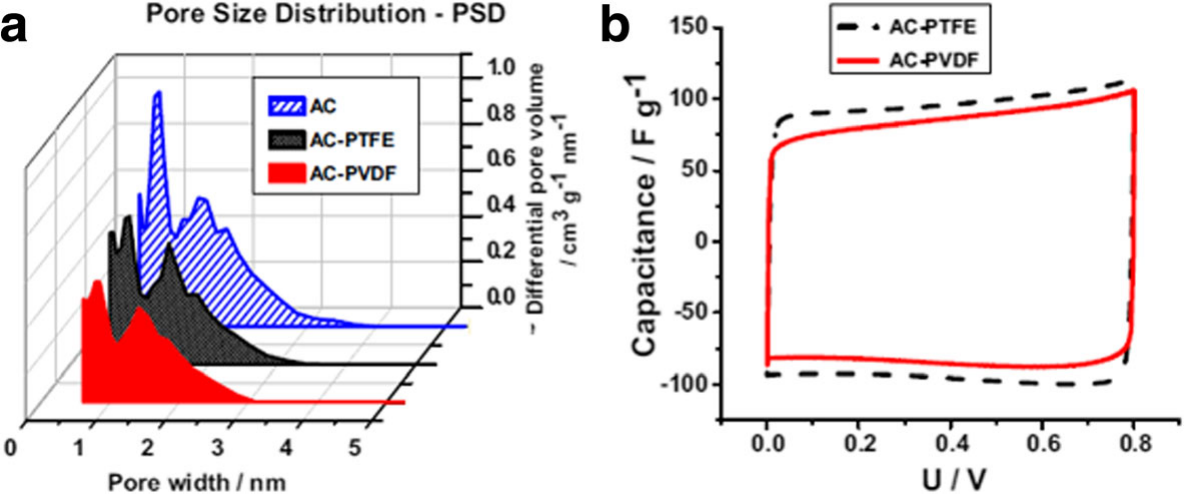
**a** Pore size distribution of AC, AC-PTFE, and AC-PVDF electrodes. **b** Cyclic voltammograms (2 mV · s^−1^) up to 0.8 V of AC/AC capacitors in 1 mol · L^-1^ NaNO3 with PTFE and PVDF binders. Reprinted with permission from [19]. Copyright 2014 Elsevier

Different types of the binders can lead to the different performances of a supercapacitor. Abbas et al. found that for activated carbon (AC), supercapacitors using the NaNO_3_ aqueous electrolyte, different binders (PTFE vs. PVDF) of the same amount (10 wt%) can affect the overall performance of the supercapacitors [19]. Electrode with PVDF binder is found less porous than the one with PTFE binder as shown in the pore size distribution in Fig. 2a. Consequently, the higher capacitance is achieved with AC-PTFE electrodes due to their higher microporous volume (pore width < 5 nm) as compared to AC-PVDF ones as shown in Fig. 2b [19].

PTFE is insulating and hydrophobic. Too much PTFE could decrease the conductivity of the electrode and inhibit the penetration of aqueous electrolyte ions into the micropores in the electrode materials, resulting in reduced energy density and specific capacitance [20]. The optimal content of PTFE depends on several factors including the active materials, electrode materials, and electrolyte, etc. Tsay et al. reported that for the carbon BP2000-based supercapacitor using the Na_2_SO_4_ solution as the electrolyte, the maximum specific capacitance and energy density are achieved using 5 wt% of PTFE in the composite. Insufficient PTFE binder could cause adhesion issue of the active materials on the electrode, and the specific capacitance and especially the capacitance retention over long cycles could also be adversely affected. In another study, Zhu et al. found that for supercapacitors made of AC on nickel foam with 6 M KOH aqueous electrolyte, the maximum specific capacitance is achieved with 10% PTFE. However, the optimal condition is different when using PVDF binders (5%) [21].

Good wettability can provide easier access for the electrolyte ions to enter the porous structure of the electrode materials and thus achieve higher double layer capacitance. To increase the hydrophilicity of PTFE binder to some aqueous electrolyte, a tiny amount of polyvinylpyrrolidone (PVP) (e.g., 3%) can be added to the PTFE dispersion [22]. Paul et al. found that the electrode material with PTFE binder has a contact angle of 151 °, which suggests a super-hydrophobicity in nature; but by adding 3% PVP, the contact angle drastically decreases to 22 ° which suggests a good wettability of the electrode [23].

**Fig. 3:**
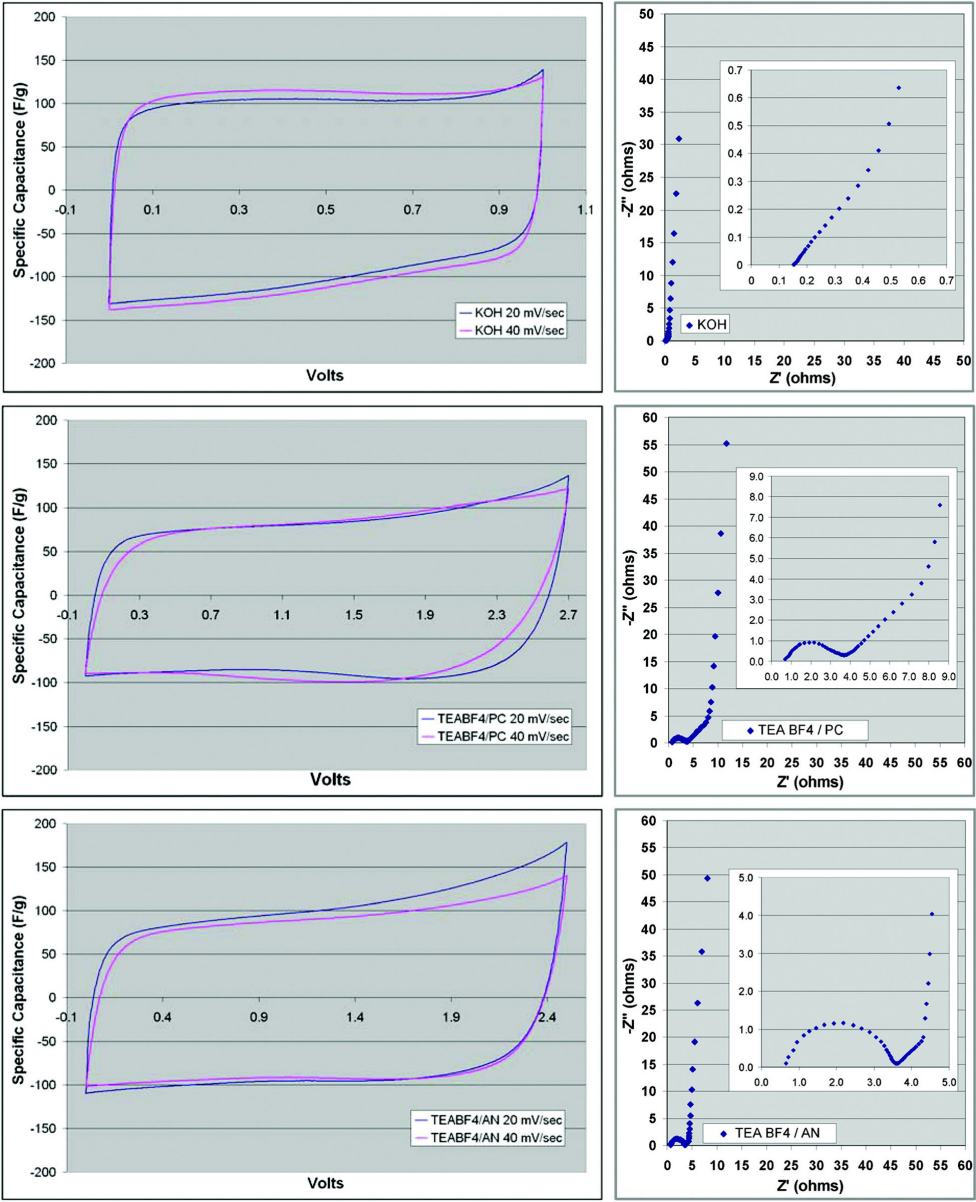
CV (*left*) and Nyquist (*right*) plots of CMG material with KOH electrolyte (*top*), TEABF_4_ in propylene carbonate (*middle*) and TEABF_4_ in acetonitrile (*bottom*). Reprinted with permission from [24]. Copyright 2008 American Chemical Society

Depending on the chosen electrolyte, the graphene-based supercapacitor can behave differently. Stoller et al. used chemically modified graphene as the active material, PTFE as the binder to form a graphene-based supercapacitor and tested it in both aqueous and organic electrolyte [24]. Figure 3 shows the different characteristics of the cyclic voltammetry (CV) and Nyquist plots of the graphene/PTFE supercapacitor using KOH, TEABF_4_ in propylene carbonate (PC), and TEABF4 in acetonitrile (AN) as the different electrolyte. It is found that aqueous electrolyte KOH provides the highest specific capacitance of 116 Fg^−1^ compared to 100 Fg^−1^ in TEABF_4_/AN, and 95 Fg^−1^ in TEABF4/PC [24]. It should also be noted that the equivalent series resistance (ESR) of the supercapacitor cells are different in these three electrolytes as suggested by the Nyquist plot. When designing a supercapacitor, one should also take into consideration of the compatibility of the electrolyte, the active materials, and the current collector.

### Graphene and Conducting Polymers Composites

**Fig. 4:**
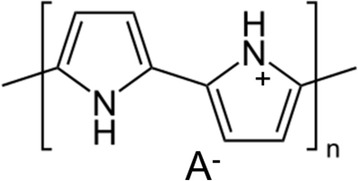
Chemical structure p-doped PPy

As discussed above, polymer binders are the very important part to form supercapacitor electrode. However, one disadvantage of using polymer binders is that they are usually not conductive and can decrease the energy density of supercapacitors. Graphene/polymer binders composites based supercapacitors are mainly based on the electrical double layer (EDL) capacitance. On the other hand, conducting polymers (CPs) offer an alternative approach and attract a lot of attention in supercapacitor applications. They are electrically conducting and can have very fast redox reaction with an electrolyte which can lead to pseudo-capacitance stored in the supercapacitor in addition to the EDL capacitance. CPs have a π-conjugated backbone which consists of single (C-C) and double (C = C) carbon bonds. Among all the conjugated CPs, poly(pyrrole) (PPy), polyaniline (PANI), and Poly(3,4-ethylenedioxythiophene) (PEDOT) are the three most commonly used types of CPs in supercapacitor applications due to their high conductivity, ease of synthesis, cost effectiveness, and light weight [25, 26].The conjugated backbones of these polymers can be p-doped by oxidation or n-doped by reduction to form p-type CP or n-type CP, respectively. Figure 4 shows chemical structure of the p-doped PPy. The positive charge is delocalized on the PPy main chain, and A^−^ represents counteranions such as NO_3_^−^, ClO_4_^−^, Cl^−^, etc.

Despite their high conductivity, fast charge and discharge, high specific capacitance as the supercapacitor electrode [27–29], the main disadvantage of using the CPs alone as the supercapacitor electrode is that they suffer stability issues over long cycles. Mechanical stress due to the volumetric change of the CPs during the redox reaction over long cycles can lead to the cracks, material loss, or even breaking of the CPs. These failures can ultimately cause a capacity drop or even break-down of the supercapacitor device over time. One solution to increase the lifetime of a CP-based supercapacitor is to compound CPs with several forms of carbons (e.g., AC, CNTs, and graphene). These composites have shown better stability since the network of carbon in the composites can adjust the volumetric change during the charge and discharge cycles and hence the capacitance retention over cycles can be greatly improved [30–32]. Generally speaking, p-doped CPs are more stable than n-doped ones [33, 34]. This session will mainly focus on the composites of p-doped CPs with graphene and its derivatives in supercapacitor applications.

#### Graphene and Polyaniline (PANI) Composites

PANI has been studied extensively as a supercapacitor electrode material due to its high conductivity, electroactivity, specific capacitance, and good stability. PANI needs a proton to be conducting and properly charged and discharged; thus, a protic solvent, an acidic solution or a protic ionic liquid, is required for PANI to be used in supercapacitor applications [35, 36]. The most common synthesis methods of PANI are oxidative polymerization and electrochemical polymerization. Other methods can also be used to produce nanostructured PANI (e.g., nanofibers and nanoparticles) such as interfacial polymerization [37], electro-spinning [38], seeding polymerizations [39], templated polymerization [40], etc. It should be noted that PANI polymers synthesized by each method often have different properties, and thus the resulting PANI-based supercapacitors can exhibit rather different performances.

**Fig. 5:**
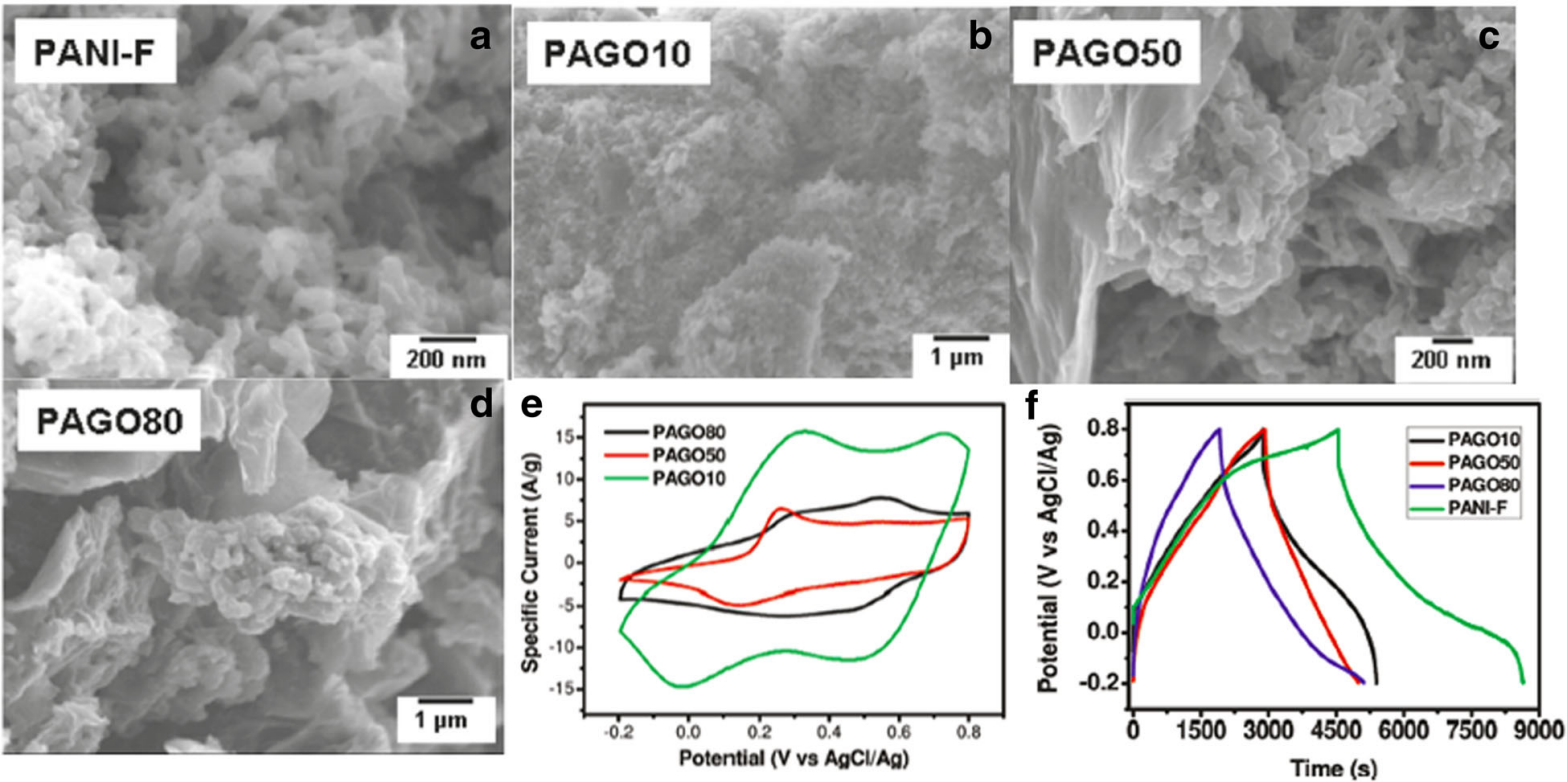
**a** SEM of pure PANI fibers (PANI-F). **b** SEM of GO and PANI composites with GO weight percentage of 10% (PAGO 10). **c** SEM of GO and PANI composites with GO weight percentage of 50% (PAGO 50). **d** SEM of GO and PANI composites with GO weight percentage of 80% (PAGO 80). **e** Cyclic voltammograms recorded in 2 M H_2_SO_4_ by using different composites coated with glassy carbon electrode as working electrode, a Pt sheet as counter electrode, and a AgCl/Ag electrode as reference electrode. The scan rate is 100 mV/s; **f** Charge/discharge cycling curves of different composite electrodes at a current density of 0.1 A/g. Reprinted with permission from [41]. Copyright 2010 American Chemical Society

Composites of PANI and graphene can be prepared by in situ polymerization of aniline with graphene suspension in acidic solution. Zhang et al. used graphene oxide (GO) to mix with PANI to form the composite and drop cast onto glassy carbon working electrode for electrochemical characterization [41]. The GO was prepared from graphite by a modified Hummers method in the study and had a layer-like structure with the size of tens of micrometers. Oxidative polymerization method was used in [41] to synthesize PANI nanofibers in 1 M aqueous HCl acidic media while ammonium peroxydisulfate ((NH_4_)_2_S_2_O_8_) was used as the oxidizing agent. As Fig. 5 shows, different GO concentrations in the GO/PANI composite lead to different composite morphologies, affecting the electrochemical behaviors as the supercapacitor electrode [41]. Pure PANI nanofibers have one pair of redox peaks in the CV curves, but the GO/PANI composites exhibit both characteristics of pure GO and pure PANI which show two pairs of the redox peaks. Pure PANI nanofibers (PANI-F) electrode has a very high specific capacitance of 420 Fg^−1^, however, the cycling stability is rather poor, and the specific capacitance reduces by almost 40% after only 5 cycles. As the GO concentration increases, the specific capacitance of the composite decreases (PAGO10: 320 Fg^−1^, PAGO50: 207 Fg^−1^, and PAGO80: 158 Fg^−1^). On the other hand, with higher GO concentration in the composites, the capacitance retention over long cycles is greatly improved, since GO nanoparticles can compensate the volumetric change of the PANI during the charge and discharge cycles.

**Fig. 6:**
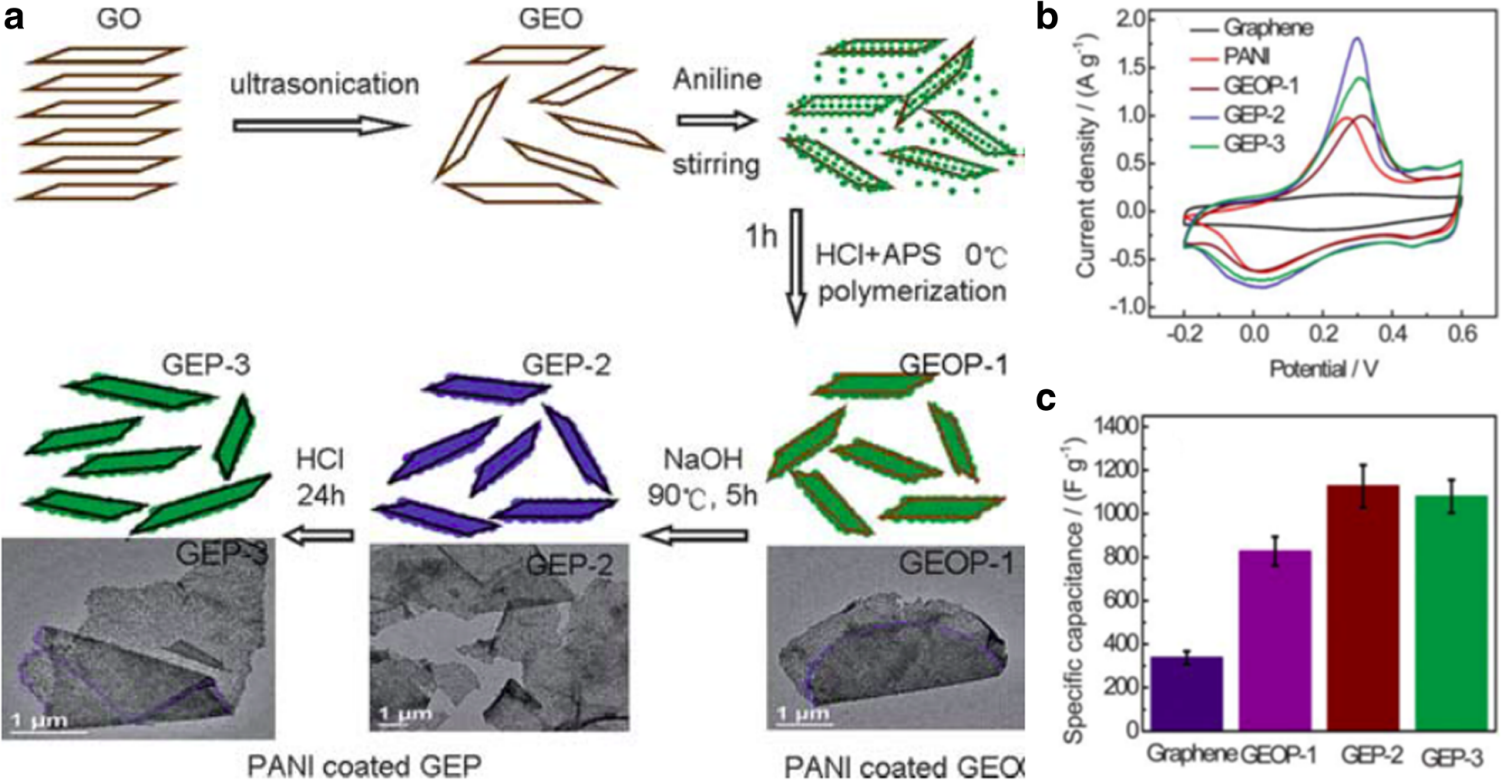
**a** A scheme illustrating the preparation process of graphene/PANI hybrid materials. **b** CV curves of graphene, PANI, GEOP-1, GEP-2, and GEP-3, at 1 mV s^−1^ in 1 M H_2_SO_4_ in the potential range from −0.2 to 0.6 V. **c** Specific capacitance changes with different samples. Reprinted with permission from [44]. Copyright 2010 Royal Society of Chemistry

GO is insulating in nature, and GO nanoparticles in the composite contribute very little to the specific capacitance. The total capacitance of the composite is mainly from the pseudocapacitance of the PANI nanofibers. Many studies [42, 43] begun to use reduced graphene oxide (RGO) to replace GO, as RGO has higher specific capacitance and can also decrease the ESR of the supercapacitor electrode due to its higher conductivity. RGO nanoparticles can be prepared by reducing the GO nanoparticles, based upon chemical, thermal, or electrochemical methods. Wang et al. used a three-step synthesis method by an in situ polymerization-reduction/dedoping-redoping process to form RGO and PANI composites [44]. This in situ process is able to facilitate a uniform dispersion of RGO into the composites, and its schematics are shown in Fig. 6a: (i) GO in ethylene glycol is ultrasonicated to get a uniform exfoliated graphene oxide suspension (GEO); (ii) Aniline solution is added to the mixture under stirring; (iii) hydrochloric acid (HCl) and ammonium persulfate (APS) are added for polymerization to form GO and PANI composites (GEOP-1); (iv) sodium hydroxide (NaOH) at 90 °C is then added to the suspension to reduce GO and dedope the PANI polymerization simultaneously to form RGO and dedoped PANI composites (GEP-2); (v) Last, HCl is reintroduced to redope PANI to form RGO and redoped PANI composites (GEP-3) [44]. The RGO and PANI composites are 30–40 nm thick and a few micrometers in size, which suggests a large specific area of these nanocomposites. It can also be seen from Fig. 6b, c that reducing GO to RGO, it results in higher redox peaks of PANI/RGO electrode and thus leads to higher specific capacitance. The highest specific capacitance in this study is GEP-2 with 1129 Fg^−1^. Similarly to GO/PANI composites, the cycling retention of RGO/PANI supercapacitor electrode is also improved compare to pure PANI electrode: GEP-2 and GEP-3 have capacitance retention of 84 and 72% after 1000 cycles, respectively.

#### Graphene and Polypyrrole (PPy) Composites

**Fig. 7:**
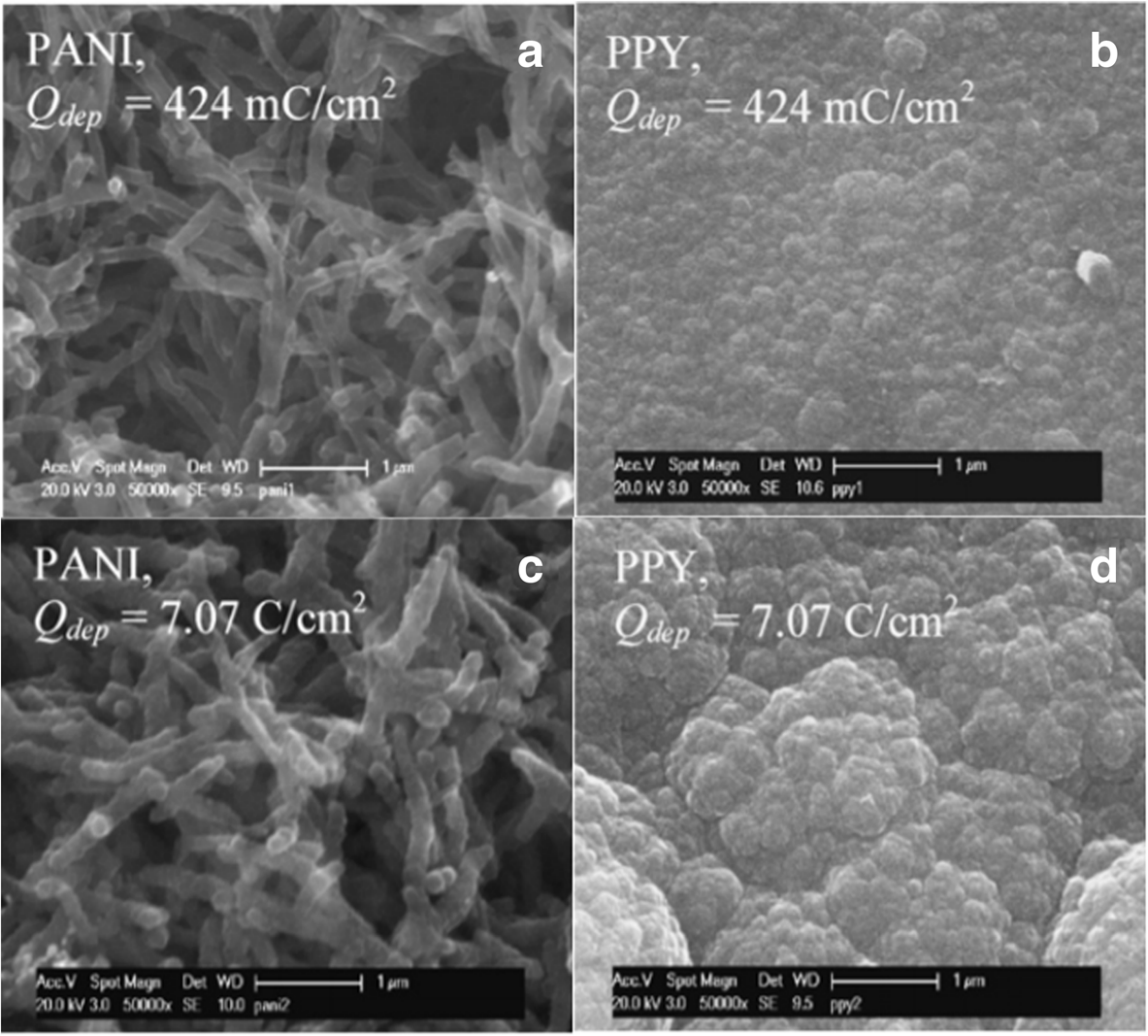
SEM images of the surfaces pure PANI and PPy films at low (424 mC/cm^2^) and high (7.07 C/cm^2^) deposition charges (Q_dep_) as indicated. Reprinted with permission from [46]. Copyright 2007 Elsevier

PPy was first demonstrated by Weiss et al. in 1963 [45]. PPy is also a very attracting CP material for supercapacitor applications since the synthesis of this polymer is simple and it has both good conductivity and thermal stability. The pure PPy polymer has very different morphology compared to PANI as Fig. 7 shows [46]. Both thin (Fig. 7a) and thick (Fig. 7c) PANI films are composed of nano-fibrils. However, the thin PPy film (Fig. 7b) which contains sub-micron particles and later forms aggregated cauliflower-like structures (Fig. 7d) with increasing polymerization charge to increase the film thickness. Such aggregated structure of the thick PPy film is not favorable for supercapacitor applications since they minimize the surface area and have the tendency to block the access of electrolyte ions. In addition, during the charge and discharge cycles, these particulate structures can easily collapse due to the stress caused by the volumetric change. Generally speaking, pure PPy film has inferior cycling performance as supercapacitors compared to PANI film [46].

Similarly to PANI/graphene nanocomposites, in situ polymerization of PPy in the presence of graphene is also preferred. By doing so, it could have better dispersion of graphene nanoparticles and minimize the aggregation. Bose et al. reduced GO in the presence of poly(sodium 4-styrenesulfonate) (Na-PSS) to modify the surface of the graphene in order to avoid the aggregation of the graphene nanosheets (GNS) [47]. The thickness of the GNS is around 2 nm, possibly double or multiple layers of single graphene sheets. After mixing surface modified GNS and pyrrole monomers in ethylene solution, ferric chloride (FeCl_3_) is added to the mixture to start the polymerization, and GNS/PPy composites are formed. A successful mix of GNS/PPy composites can be represented by checking the presence of both peaks of pure GNS and PPy in the Raman spectrum. In [47], it has been observed that the GNS/PPy composites have almost twice of the specific capacitance and much better cycling performance as supercapacitor electrode compared to pure PPy film. Again, these results provided evidence that the incorporation of graphene into PPy can facilitate electrochemical utilization of PPy and provided mechanical support to PPy to enhance the structural stability of the composites during the charge and discharge cycles.

#### Graphene and Poly(3,4-ethylenedioxythiophene) (PEDOT) Composites

Poly(3,4-ethylenedioxythiophene) (PEDOT) was firstly developed in the 1980s by scientists at Bayer AG research labs in Germany [48]. PEDOT can be prepared using standard oxidative chemical or electrochemical polymerization methods, and it has very high conductivity from a few to 500 S/cm at its doped state [49, 50]. PEDOT was first found insoluble, but later this solubility issue was circumvented by using a water-soluble polyelectrolyte, poly(styrene sulfonic acid) (PSS) [51]. PEDOT also has a wide potential window, good thermal and chemical stability which attract much attention from supercapacitor community. Compared to other conducting polymers, PEDOT has excellent cycling stability, with 80% capacitance retention over 70,000 cycles [52]. However, one disadvantage of PEDOT as supercapacitor is the large molecular weight which leads to a relatively low specific capacitance [36].

**Fig. 8:**
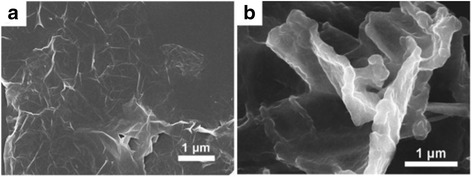
SEM images of (**a**) pure RGO film (**b**) RGO, and PEDOT composites film. Reprinted with permission from [57]. Copyright 2013 Royal Society of Chemistry

In order to minimize the aggregation of graphene nanoparticles in the composite, similar in situ polymerization method can be used to form the graphene/PEDOT composites. First, PSS and ethylene dioxythiophene (EDOT) monomers are mixed in an aqueous solution such as HCl [53], DI water [54, 55], sometimes the degassing process is performed [55, 56] before dispersing graphene or graphene derivative nanoparticles into the resultant solution with stirring or sonication. Next, oxidants such as ammonium peroxydisulfate [(NH_4_)_2_S_2_O_8_)] and iron (III) chloride (FeCl_3_) [53], or sodium persulfate (Na_2_S_2_O_8_) and iron (III) sulfate [Fe_2_(SO_4_)_3_] [56] are then added to initiate the polymerization and finally form the graphene/PEDOT composites. Figure 8 shows the SEM images comparison of RGO and RGO/PEDOT composites [57]. Original RGO films are relatively large and smooth, while the RGO/PEDOT composite film formed a curved planar film of around 200 nm thick [57]. With the incorporation of graphene, it is reported that the electrical conductivity of graphene/PEDOT was improved more than two times than that of a pristine PEDOT, while the mechanical strength was also shown sixfold enhancement at the same time [58]. The role of graphene in the composites is furnishing pathways for charge percolation and propagation, thus improving the overall charge transport behavior of PEDOT [59].

Pristine PEDOT supercapacitors have a specific capacitance in the range from 70 to 130 Fg^−1^ [52, 60, 61] depending on the different polymerization methods. However, both enhanced specific capacitance and cycling stability performance have been observed with graphene/PEDOT composites-based supercapacitors. For example, Alvi et al. reported supercapacitors based on graphene/PEDOT with a specific capacitance of 304 and 261 Fg^−1^ in HCl and H_2_SO_4_ electrolytes, respectively [53]. Wen et al. reported that GO/PEDOT composite electrode has a specific capacitance of 136 Fg^−1^, and RGO/PEDOT composite electrode has a specific capacitance of 209 Fg^−1^ with 87% capacitance retention over 2000 cycles [62]. The roles of graphene in the composites as supercapacitors are (i) it forms a heterogeneous structure with PEDOT, which effectively reduces the structural damages (i.e., collapse, peeling off, and cracking) caused to PEDOT due to volumetric change (i.e., swelling, and shrinkage) of PEDOT during the charge-discharge cycles, (ii) graphene or RGO has a higher electrical conductivity than PEDOT: PSS, making the composite more conductive, and (iii) graphene addition makes the composite has a 3D morphology which can lead to significant improvement on specific capacitance by providing large surface area for electrolyte penetrations and redox reactions [60].

#### Graphene and Conducting Polymers (CPs) Composites Comparison and Summary

Graphene and its derivatives can have great influence on the morphologies, electrical properties, and the structural stabilities of the CPs when they are made into composites, and thus lead to drastic enhancement of the electrochemical properties of CPs. However, for a graphene/CPs supercapacitor, there are many factors which can determine the ultimate supercapacitor performance (i.e., specific capacitance, cycling stability, charge-discharge properties, etc.) such as polymerization method, intrinsic properties of individual CPs, electrolyte, dispersion/aggregation of the graphene in the composites, different properties of graphene used, etc. Generally speaking, for the aforementioned three types of CPs (PANI, PPy, and PEDOT), there are distinct advantages and disadvantages of using individual CPs to form the composite. PEDOT has the largest molecular molar mass, hence, the specific capacitance of the graphene/PEDOT composites supercapacitor is normally smaller than the ones formed with PANI or PPy. On the other hand, the larger particles formed in the PPy film make it less porous than PANI or PEDOT, thus the cycling performance of PPy is normally the worst among these three CPs. In addition, the conductivity comparison of these CPs is PEDOT > PPy > PANI [63], which can affect the ESR of the supercapacitors.

**Fig. 9:**
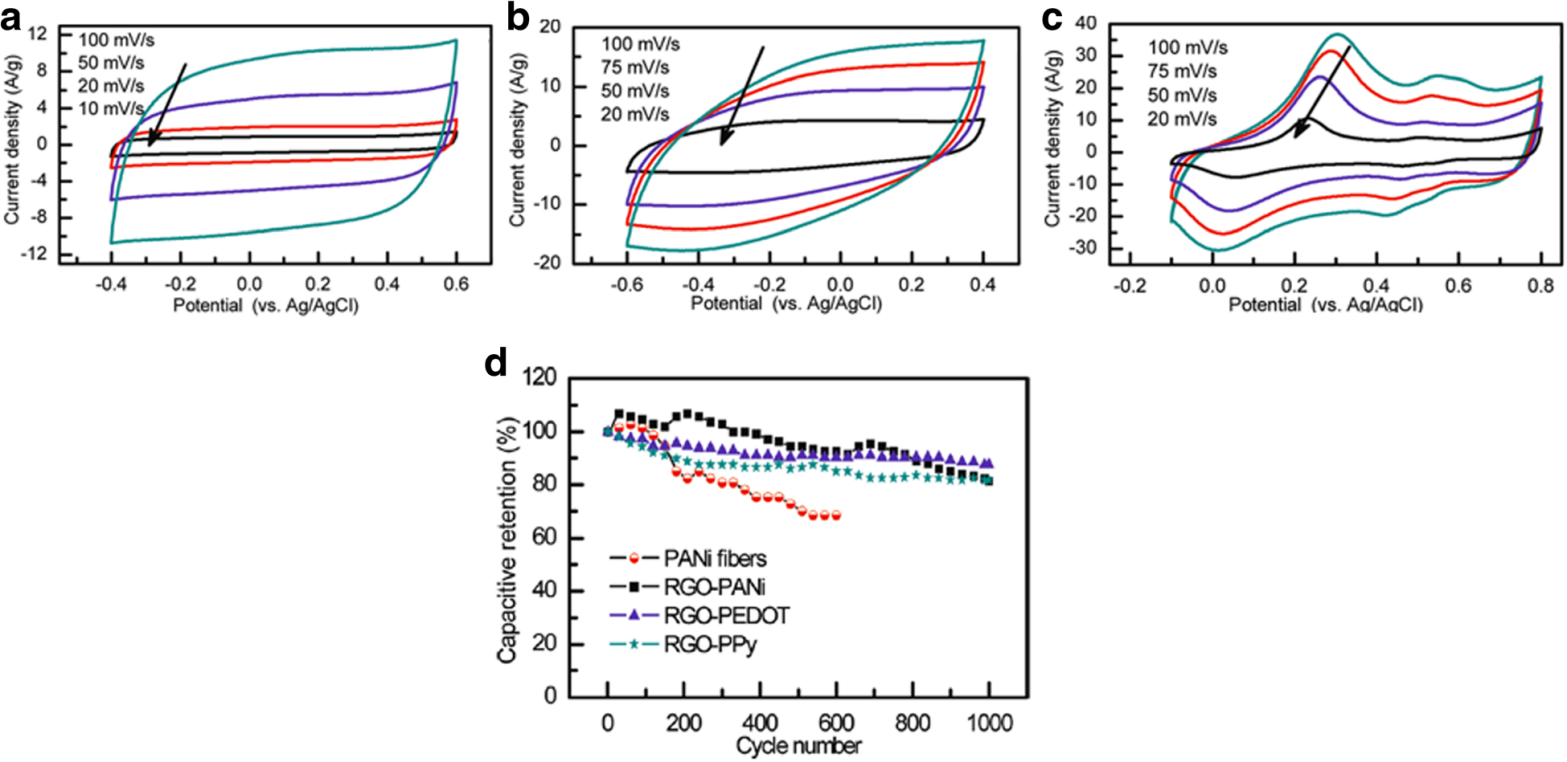
Cyclic voltammograms of (**a**) RGO-PEDOT (**b**) RGO-PPy, and **c** RGO-PANi cycle stability of PANi fibers, RGO-PANi, RGO-PEDOT, and (**d**) RGO-PPy during the long-term charge/discharge process. Reprinted with permission from [42]. Copyright 2012 American Chemical Society

Zhang et al. compared the supercapacitor performance of the composites formed of RGO nanosheets with PANI, PPy, and PEDOT polymers using similar polymerization and mixing methods [42]. The specific capacitances of the supercapacitor electrodes from RGO/PANI, RGO/PPy, and RGO/PEDOT composites are 361, 249, and 108 Fg^−1^ at the current density of 0.3 Ag^−1^, respectively. Figure 9a–c shows the different cyclic voltammograms (CV) of the RGO and CPs composite electrodes at different scan rates [42]. The quasi-rectangular CV curves of both RGO-PEDOT and RGO-PPy suggest good capacitive behaviors of both supercapacitor electrodes. The redox peaks in the range of +0.3 to 0 V on the RGO/PANI CV curves are due to the redox transition of PANI between the semiconducting state form and the conductive form [42, 64, 65]. Figure 9d shows the cycle stability of PANI fibers, RGO-PANI, RGO-PEDOT, and RGO-PPy during the long-term charge/discharge process [42]. With the RGO addition, all the composite supercapacitor electrodes showed good cycling performance compared to pristine PANI fibers with capacitance retention of only 68% after 600 cycles. However, RGO/PEDOT has the best capacitance retention of 88% after 1000 cycles, while RGO/PANI and RGO/PPy have the similar cycling performance of 82 and 81% retention after 1000 cycles [42].

**Fig. 10:**
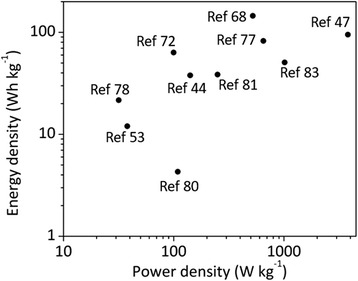
Ragone plot of graphene and CP composites supercapacitors in the summarized references listed in Table 1

Table 1 summarizes the recent development of graphene and CP (PANI, PPy, and PEDOT) composites in supercapacitor applications. Ragone plot comparing the power density and energy density of the graphene and CP composites supercapacitors in the summarized references is shown in Fig. 10. It should be noted that most studies reported specific capacitance of supercapacitor electrode based on three-electrode system (i.e., working, counter, and reference electrodes). Symmetric supercapacitor cells composed of anode and cathode of the same materials normally have less than half the specific capacitance of single electrode considering the separator and electrolyte weight. In addition, it is not surprising that very high specific capacitances in some studies were reported since only a small amount of active materials were applied/coated on the working electrode for electrochemical characterization. For practical purposes, it is hard to achieve the same level of specific capacitance since it may not increase linearly with increasing amount of materials.

**Table 1:** Comparison of the supercapacitor performances based on graphene/conducting polymers composite materials

Years	Materials	Setup (no. of electrodes)	Electrolyte	Specific capacitance	Capacitance retention	Energy/power density	ESR	Refs
2009	Graphene/PANI	3	1 M H_2_SO_4_	408 Fg^−1^ from CV at a scan rate of 5 mVs^−1^	84% after 40 cycles	NA	NA	[92]
2009	Graphene/PANI	3	1 M H_2_SO_4_	233 Fg^−1^	NA	NA	0.5–11.6 Ω	[93]
2010	GO/PANI RGO/PANI	3	1 M H_2_SO_4_	GO/PANI: 827 Fg^−1^ from CV at a scan rate of 1 mVs^−1^ RGO/PANI: 1129 Fg^−1^ from CV at a scan rate of 1 mVs^−1^	GO/PANI: 59% after 1000 cycles RGO/PANI: 84% after 1000 cycles	RGO/PANI: 37.9 Wh kg^−1^ at 141.1 Wkg^−1^	NA	[44]
2010	GO/PANI RGO/PANI	3	2 M H_2_SO_4_	320 Fg^−1^ at 0.1 Ag^−1^ 480 Fg^−1^ at 0.1 Ag^−1^	NA (cycling test was only 5 cycles)	NA	2 Ω	[41]
2010	Graphene/PANI	3	1 M H_2_SO_4_	1046 Fg^−1^ from CV at a scan rate of 1 mVs^−1^	NA	145 Wh kg^−1^ at 522 Wkg^−1^	NA	[94]
2012	RGO/PANI	3	NA	361 Fg^−1^ at 0.3 Ag^−1^	82% after 1000 cycles	NA	NA	[42]
2013	Graphene/PANI paper	3	1 M H_2_SO_4_	763 Fg^−1^ at 1 Ag^−1^	82% after 1000 cycles	NA	4.1 Ω	[72]
2013	RGO/PANI	3	1 M H_2_SO_4_	257 Fg^−1^ at 0.1 Ag^−1^	98% after 1000 cycles	NA	0.5 Ω	[95]
2014	GO/PANI	3	0.5 M H_2_SO_4_	448 Fg^−1^ at 0.5 Ag^−1^	81% after 5000 cycles	NA	3.67 Ω	[96]
2017	Crumpled graphene/CNT/PANI	2	KOH	456 Fg^−1^ at 0.1 Ag^−1^	97% after 1000 cycles	63.3 Wh kg^−1^ at 100 Wkg^−1^	~1.2 Ω	[97]
2017	RGO/PANI	2	1 M H_2_SO_4_	438.8 Fg^−1^ at 0.5 Ag^−1^	76.5% after 2000 cycles	NA.	NA	[98]
2010	Graphene/PPy	2	1 M NaCl	165 Fg^−1^ at 1 Ag^−1^ at end of 1000th cycle	NA	NA	NA	[99]
2011	GO/PPy	3	2 M H_2_SO_4_	417 Fg^−1^ from CV at a scan rate of 100 mVs^−1^ 267 Fg^−1^ from CV at a scan rate of 100 mVs^−1^	90% after 500 cycles	95 Wh kg^−1^ at 3797 Wkg^−1^	NA	[47]
2012	RGO/PPy	3	NA	249 Fg^−1^ at 0.3 Ag^−1^	81% after 1000 cycles	NA	NA	[42]
2012	RGO/PPy	3	1 M H_2_SO_4_	420 Fg^−1^ at 0.1 Ag^−1^ 240 Fg^−1^ at 5 Ag^−1^	93% after 200 cycles	NA	~2 Ω	[100]
2014	RGO/PPy nanowire	3	1 M KCl	728 Fg^−1^ at 0.5 Ag^−1^ 675 Fg^−1^ at 2.5 Ag^−1^	93% after 1000 cycles	NA	0.19 Ω	[101]
2014	Exfoliated graphene/PPy	3	3 M KCl	351 Fg^−1^ at 1 Ag^−1^	91% after 1000 cycles	65.1 Wh kg^−1^ at 13 Wkg^−1^ 82.4 Wh kg^−1^ at 650 Wkg^−1^	~0.2 Ω	[102]
2016	Graphene/PPy nanotubes	3	Polyvinyl alcohol (PVA)/H_2_SO_4_ hydrogel	514 Fg^−1^ at 0.2 Ag^−1^ 420 Fg^−1^ at 1 Ag^−1^	95% after 1500 cycles	21.6 Wh kg^−1^ at 31.7 Wkg^−1^	NA	[103]
2016	RGO/PPy	2	1 M H_2_SO_4_	440 mFcm^−2^ at 0.5 mAcm^−2^	71-80% after 5000 cycles	31.3 μWh cm^−2^ at 1.2 mW cm^−2^	3.5–5.5 Ω	[104]
2016	Sulfonated graphene/PPy	3	1 M KCl	310 Fg^−1^ at 0.3 Ag^−1^	71% after 1500 cycles	4.3 Wh kg^−1^ at 109 Wkg^−1^	0.6 Ω	[105]
2011	Graphene/PEDOT	3	2 M HCl 2 M H_2_SO_4_	HCl: 304 Fg^−1^ from CV at a scan rate of 10 mVs^−1^ H_2_SO_4_: 261 Fg^−1^ from CV at a scan rate of 10 mVs^−1^	NA	12 Wh kg^−1^ at 38 Wkg^−1^	~0.4 Ω	[53]
2012	RGO/PEDOT	3	NA	108 Fg^−1^ at 0.3 Ag^−1^	88% after 1000 cycles	NA	NA	[42]
2013	Graphene/PEDOT	3	1 M H_2_SO_4_	270 Fg^−1^ at 1 Ag^−1^	93% after 10000 cycles	38.5 Wh kg^−1^ at 250 Wkg^−1^	NA	[106]
2014	RGO/PEDOT	3	1 M H_2_SO_4_	213 Fg^−1^ at 0.5 Ag^−1^	87% after 2000 cycles	NA	<2 Ω	[62]
2016	Hallow RGO/PEDOT	2	PVA/H_3_PO_4_ gel electrolyte	304.5 mFcm^−2^ at 0.08 mAcm^−2^	96% after 10000 cycles	27.1 μWh cm^−2^ at 66.5 μW cm^−2^	306.8 Ω	[107]
2017	RGO/PEDOT:PSS	2	6 M KOH	367 Fg^−1^ at 1 Ag^−1^	80% after 30,000 cycles	50.7 Wh kg^−1^ at 1014 Wkg^−1^	1.95 Ω	[108]

*NA* not available

### Graphene and Polymer Composites for Flexible Supercapacitor Applications

**Fig. 11:**
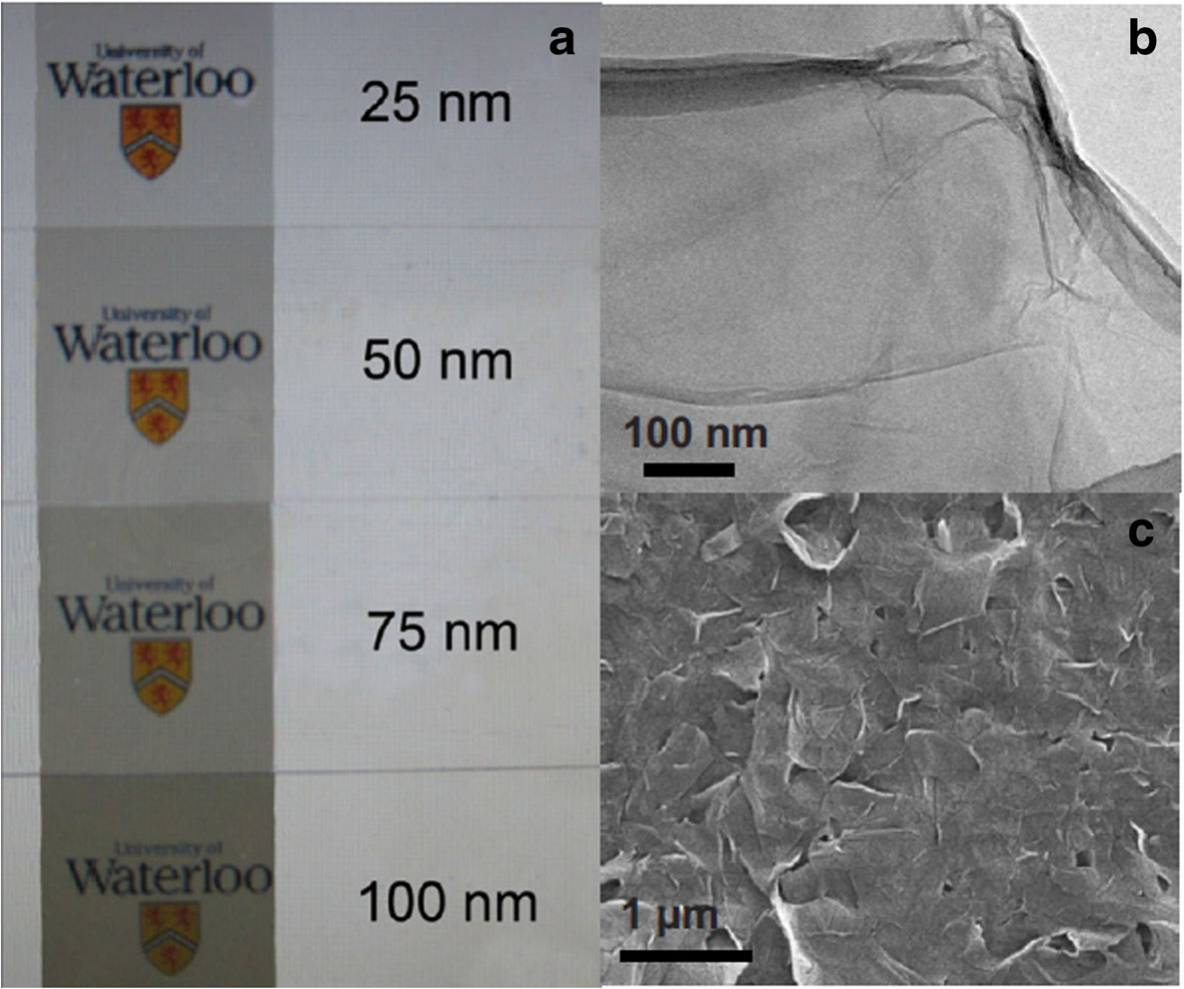
**a** Photographs of transparent thin-films of varying thickness on glass slides. **b** TEM image of graphene collected from dispersion before filtration. **c** SEM image of 100 nm graphene film on glass slide. Reprinted with permission from [76]. Copyright 2012 AIP Publishing LLC

The recently developed flexible electronic devices such as flexible displays, curved smartphones, flexible implantable medical devices, and wearable electronic devices imply that flexible devices are beginning to emerge as the leading revolution in next generation of electronics. Several advantages of flexible electronic devices compared to conventional electronic devices include lighter weight, wearability, bendability, environmental friendliness, reduced cost, etc. In order to match the fast growth of flexible electronic devices, energy storage systems that are light, thin, and flexible should also be developed. Recently, considerable attempts have been made to develop flexible supercapacitor electrode based on carbon materials including activated carbon [7, 66], carbon nanofibers [67, 68], carbon nanotubes [69–71], and graphene [72–75]. Graphene film can be prepared ultrathin (<100 nm) by filtration method and transferred to a flexible polyethylene terephthalate (PET) substrate to make flexible supercapacitor electrodes as Fig. 11 shows [76]. Although the PET substrate is flexible and can provide mechanical support to the graphene film, it does not provide any capacitance as supercapacitors, thus the device capacity would be affected by introducing the additional PET substrate. In addition, there are issues associated with the transferring process, and it is hardly a scalable process as wrinkles or other defects can happen during the graphene transfer process. Furthermore, the restacking of graphene can cause the decrease of the surface area which leads to a relatively specific capacitance of 100 Fg^-1^ for the 100-nm-thick graphene film. There are other types of flexible supercapacitor electrodes based on graphene materials, such as graphene paper [77, 78], graphene foam [79, 80], and graphene on carbon cloth/fabric [7, 81–83]. However, in each case, there may exist issues in graphene restacking, yield, limited power density and energy density, cost, and scalability. Similar to existing activated carbon supercapacitor industry, roll-to-roll manufacturing compatibility is an ideal feature for the flexible supercapacitor materials.

**Fig. 12:**
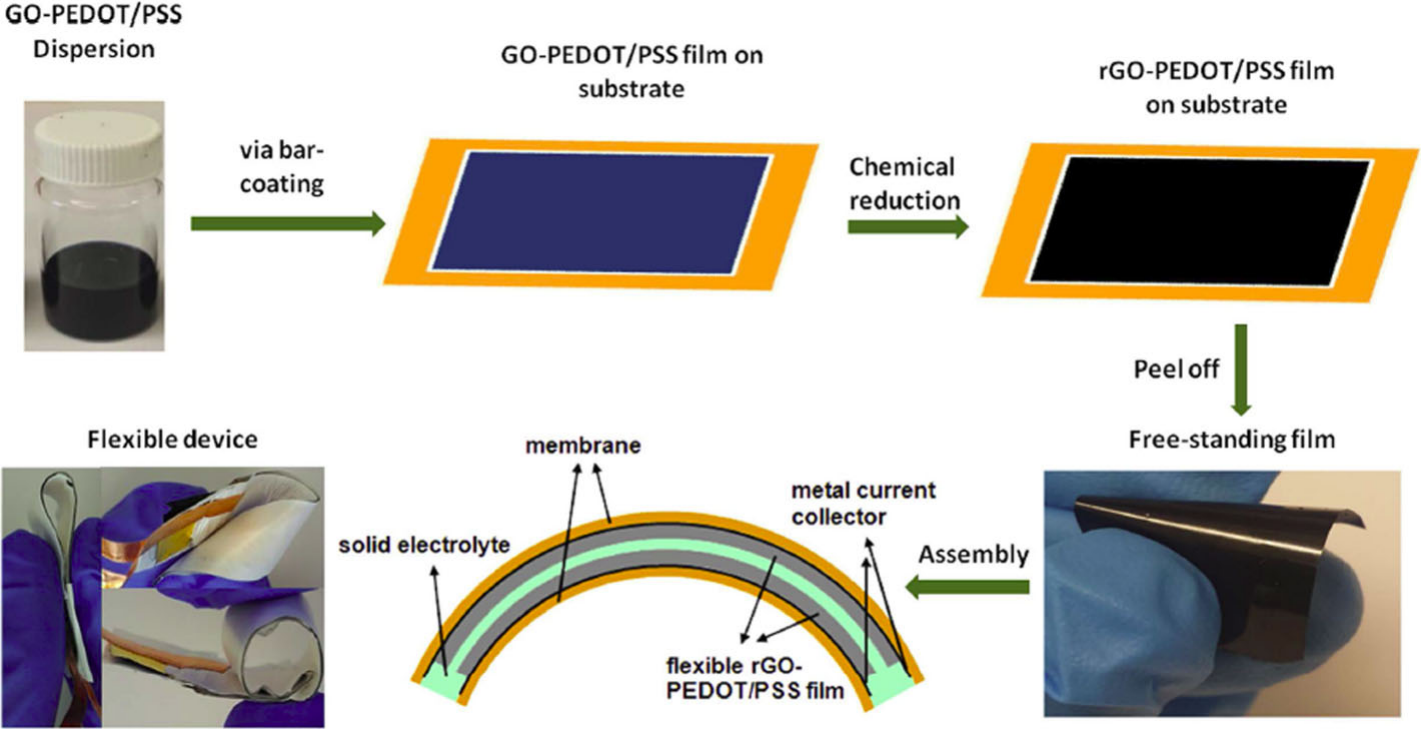
Schematic illustration of the preparation process of rGO-PEDOT/PSS films and the structure of assembled supercapacitor devices. Reprinted with permission from [84]. Copyright 2015 Nature Publishing Group

Graphene/conducting polymers (CPs) composite film, due to its flexible nature, becomes a viable option for flexible supercapacitor applications as it can also adapt to the roll-to-roll manufacture. Graphene/CPs film can be assembled with solid electrolyte to form a flexible supercapacitor device which can be subject to high degrees of bending or twisting without losing the device integrity. Meanwhile, as it is discussed previously, CP itself can provide pseudocapacitance due to the redox reaction, increasing the specific capacitance, as well as the capacity of the device. Figure 12 shows the schematics of a typical assembly process of a flexible supercapacitor using RGO-PEDOT/PSS film [84]. The PVDF substrate used in the coating process was later peeled off, which is a good way to eliminate the unnecessary weight and volume of the whole supercapacitor device. Poly(vinyl alcohol) (PVA)/H_3_PO_4_ gel was used as the solid electrolyte in this device, and gold was sputter coated on one side of the electrode as the metal current collector [84]. Polymer gel electrolyte can be used as both a porous separator and electrolyte reservoir because of its microchannels or pores inside the structure, which facilitate the flow of electrolyte ions and avoid electrical shorting between the electrodes. Furthermore, the semi-solid framework of gel electrolyte provides good adhesion with minimal distance between the electrode/electrolyte/electrode interfaces, and thus efficiently enhances the charge-storage mechanism [85].

**Fig. 13:**
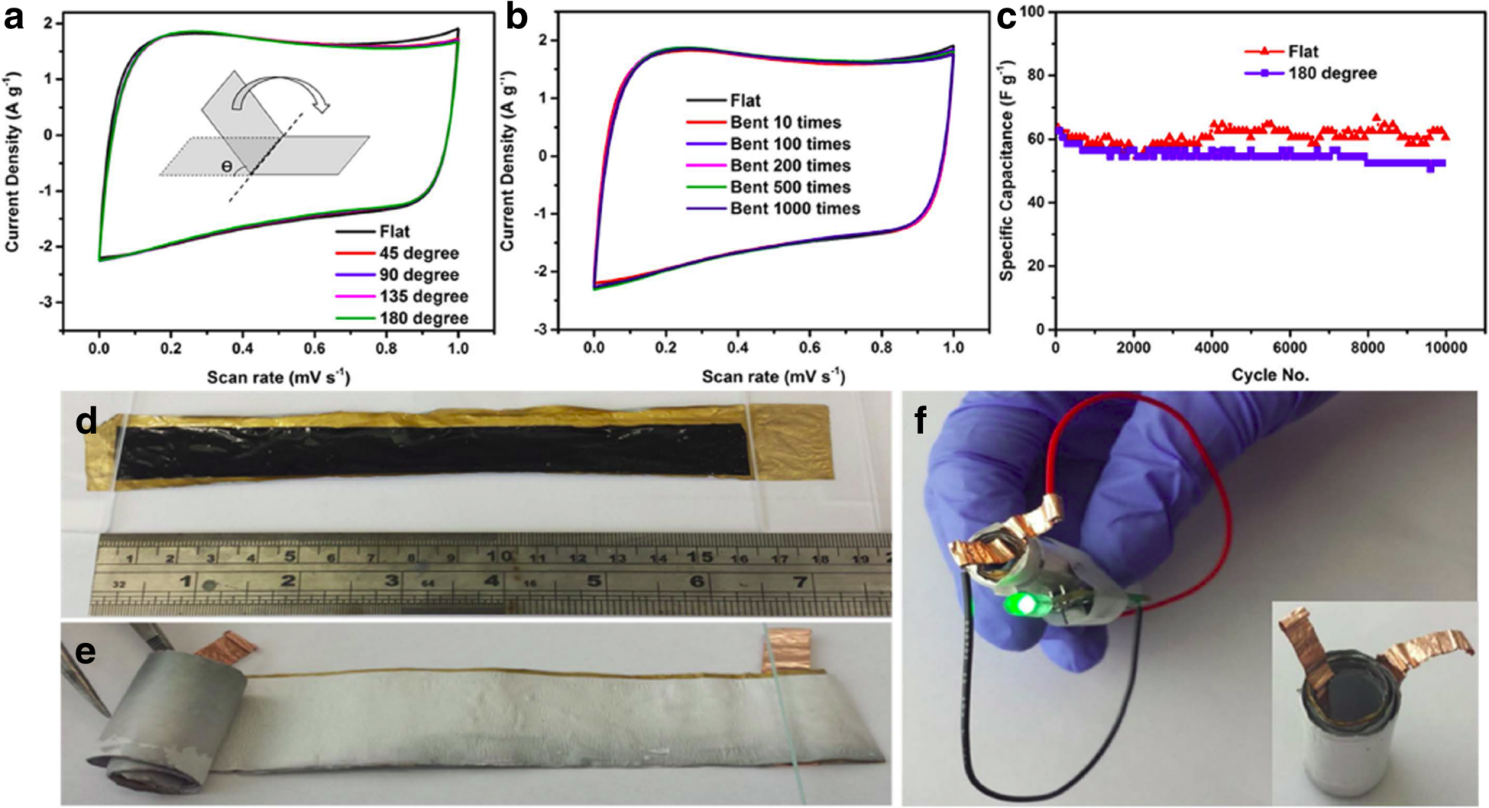
**a** CVs of rGO-PEDOT/PSS during bending. Scan rate = 50 mV s^−1^. **b** CVs of rGO-PEDOT/PSS after being subject to bending. **c** Long-term test of rGO-PEDOT/PSS under flat or 180 ° bended states at a current density of 1 A g^−1^. **d** Flexible films coated with solid electrolyte spread out on an Au-coated membrane, **e** rolled design, and **f** the resulting device used to power a green light-emitting diode (LED). Reprinted with permission from [84]. Copyright 2015 Nature Publishing Group

Bending test is usually necessary to examine the structural and electrical integrity of the flexible supercapacitor device subject to different bending angles and cycles. Bending or twisting can cause stress on the films which may lead to material loss, material fatigue (e.g., crack) or even material failure (e.g., fracture). CV and/or charge-discharge curves are monitored at several of bending angles during the bending cycles [84, 86–90] to test the device integrity. Graphene, due to its excellent mechanical strength, can prevent the flexible device from being ruptured by the repeatedly applied loads during the bending/twisting test, and it becomes an important part in the fabrication of the flexible electrode. Figure 13 shows the CV and specific capacitance of RGO-PEDOT/PSS flexible supercapacitor during the bending test [84], and it should be noted from Fig. 13b that there is no significant change of CV curves after 1000 bending cycles with a large bending angle (at 180 °). Figure 13c suggests that the specific capacitance of device in the 180 °bended state is only 5% smaller compared to the one under flat state after 10000 charge and discharge cycles. The flexible supercapacitor device made from a long strip of electrodes (15 × 2 cm) that have been rolled up as shown in Fig. 13d, e) was powerful enough to power a light-emitting diode for 20 s when fully charged (Fig. 13f) [84].

## Conclusions

The emergence of graphene has undoubtedly changed the scope of supercapacitor field due to its outstanding electrochemical properties along with other unique properties such as large surface area, high electrical conductivity, light weight, and mechanical strength. The recent development of graphene and polymer composites showed very promising features of these composite materials for supercapacitor applications. Compounded with binder polymers, graphene would compensate the undesired features of insulating polymers (e.g., insulating nature, low surface area, and low specific capacitance). On the other hand, when graphene makes composites with conducting polymers, it would provide mechanical support for the framework of the polymers and thus greatly improve the cycling performance as well as the specific capacitance. In addition, the flexible nature of the graphene/polymer film makes it possible for flexible, wearable, conformable energy storage devices. Despite the innovative ideas and techniques that have been demonstrated for the graphene/polymer supercapacitor device with unique features not possessed by current state-of-art technology, there remain a lot of challenges for graphene/polymer composite-based supercapacitors to reach their full potentials. One of the main challenges is to find a feasible way for the low-cost mass production of graphene/polymer supercapacitor electrode without compromising the micro/nanostructures of graphene due to the restacking or aggregation. Many studies have demonstrated the compatibility of large-scale coating or roll-to-roll manufacture capability of graphene/polymer supercapacitor electrodes, however, there is still a need to have a rational design of the porous structures inside the supercapacitor electrodes to form hierarchical interconnected porous microstructures and avoid the formation of dead volume or the collapse of the porous microstructures [91]. Furthermore, in most studies related to graphene/polymer-based supercapacitors, the mass specific capacitance of the supercapacitor electrode is often emphasized as one of the most important features. However, these values are often derived from the weight of a small amount of active material applied on the working electrode. In practical supercapacitor devices, to obtain a reasonable value of the device capacity, thicker coating or larger amount of material are often needed. It should be noted that the electrode total capacitance does not increase linearly with increasing amount of materials. In addition, one should also take into consideration the weight of current collector, electrolyte, and separator for supercapacitor device perspective. These aspects should be all considered to develop a deeper understanding of storage mechanism, interfacial relation, and designs of graphene/polymer-based supercapacitors. Last but not the least, integration of graphene/polymer supercapacitors with other electronic devices (e.g., solar cells, batteries) remains a challenge for practical applications. In conclusions, graphene/polymer composites have great potential for supercapacitor applications to improve current activated carbon-based supercapacitors, and we believe graphene/polymer-based supercapacitor would find its place in commercialization in the near future.
